# Comprehensive profiling of humoral immune responses to pan-respiratory pathogens: Insights from diverse cohorts

**DOI:** 10.1016/j.isci.2026.117050

**Published:** 2026-07-31

**Authors:** Xuanming Xu, Hui Wang, Lian Tang, Peiru Nie, Fengzhou Yang, Yajie Wang

**Affiliations:** 1 National Key Laboratory of Intelligent Tracking and Forecasting for Infectious Diseases, Beijing Key Laboratory of Viral Infectious Diseases, Department of Clinical Laboratory, Beijing Ditan Hospital, Capital Medical University, Beijing 100015, China; 2 Department of Neurology, Beijing Tiantan Hospital, Capital Medical University, Beijing 100070, China; 3 Department of Clinical Laboratory, Peking University Ditan Teaching Hospital, Beijing 100015, China

**Keywords:** Respiratory pathogens, Protein microarray, Humoral immunity, IgG reactivity, Post-pandemic era

## Abstract

The COVID-19 pandemic profoundly disrupted respiratory pathogen circulation and reshaped population-level humoral immunity. Using a high-throughput pan-respiratory pathogen protein microarray containing 56 antigens, we profiled serum immunoglobulin G (IgG) responses in 287 individuals across pre-pandemic, COVID-19, and post-pandemic periods, as well as validated the findings in an independent cohort of 175 participants. SARS-CoV-2 exposure induced strong cross-reactivity with SARS-CoV but minimal reactivity to MERS-CoV, while variant-specific immunity progressively shifted from pre-Omicron strains toward Omicron lineages. Spike-specific IgG declined over time, whereas nucleocapsid-specific IgG remained relatively stable. Immune responses to common respiratory pathogens were also broadly reorganized, with decreases in human coronaviruses, influenza B, respiratory syncytial viruses, and human parainfluenza viruses, stable influenza A responses, and increased human metapneumovirus-specific IgG. These findings define a post-pandemic humoral immune landscape and support sustained serological surveillance to identify immunity gaps, anticipate changing infection susceptibility, and strengthen preparedness for future respiratory epidemics.

## Highlights

• A high-throughput pan-respiratory microarray profiled immunity across three periods
• SARS-CoV-2 exposure induced cross-reactivity with SARS-CoV but little with MERS-CoV
• Post-pandemic spike IgG declined, nucleocapsid IgG persisted, immunity shifted to Omicron
• IgG responses to various respiratory pathogens showed divergent post-pandemic patterns

## Graphical abstract

**Figure:**
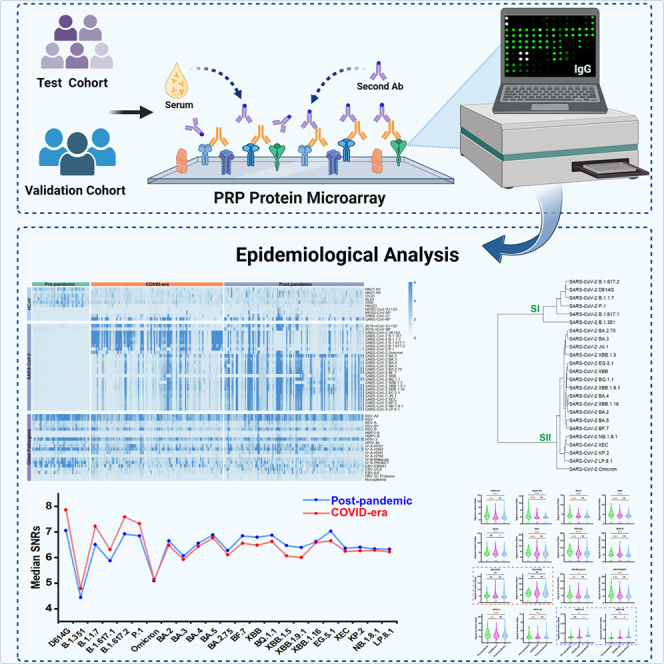

## Teaser

Health sciences; Virology

## Introduction

In recent years, epidemic prevention and control have become challenging owing to the appearance of SARS-CoV-2, significantly increasing the public health burden related to respiratory infections.^1^^,^^2^ Despite the most severe phases of COVID-19, the ongoing evolution of SARS-CoV-2, driven by the emergence of variants of concern (VOCs) and variants of interest (VOIs), underscores the remarkable adaptability of this virus.^3^ The waves of SARS-CoV-2 infections continue to appear, particularly novel variants/lineages (such as JN.1, KP.8, and XEC) that may exhibit increased transmissibility, virulence, or immune escape.^4^ The implementation of non-pharmaceutical interventions (NPIs) (such as lockdowns, the use of facemasks, and social distancing), along with repeated infections and vaccine-induced immunity, has noticeably changed the global respiratory disease landscape, resulting in an enduring immunological impact.

The Betacoronavirus family comprising three highly pathogenic coronaviruses (HPCoVs), namely, SARS-CoV, MERS-CoV, and the recently emerged SARS-CoV-2, causes severe respiratory disease in humans.^5^^,^^6^ Notably, the sequence homology of the spike (S) protein between SARS-CoV-1 and SARS-CoV-2 is approximately 77%, whereas that between MERS-CoV and SARS-CoV is only 32%, indicating a high potential for immune cross-reactivity primarily between the two SARS lineages.^7^ Indeed, multiple studies have demonstrated that repeated SARS-CoV-2 infection and vaccination elicit serological cross-reactive binding antibodies against the S protein.^8^^,^^9^^,^^10^^,^^11^ These findings reveal that the human antibody profile against coronaviruses has been profoundly reshaped and broadened in the post-pandemic era, which is crucial for the development of vaccines and the assessment of the risk of potential sarbecovirus pandemics.

In the COVID-19 era, vaccination strategies and strict NPIs have led to a decline in the circulation of other respiratory pathogens, raising concerns about immunity gaps against seasonal influenza viruses (IVs), common coronaviruses, and other respiratory infections.^12^^,^^13^^,^^14^ Recent epidemiological surveys revealed that following the relaxation of COVID-19 control measures, IVs and respiratory syncytial virus (RSV) restored their typical circulation levels during the autumn and winter.^15^^,^^16^^,^^17^ Meanwhile, there is an increasingly complex situation regarding respiratory diseases, which manifests as the coexistence of one disease with multiple infections. A comprehensive analysis of more than 2 million global SARS-CoV-2 samples revealed that coinfections accounted for approximately 0.35% of the observed cases.^18^ In addition, several hospital surveys have shown that *Mycoplasma pneumoniae* (MP) cases frequently involve coinfections with IVs or RSV.^19^^,^^20^^,^^21^ Compared with single infections, coinfections are generally associated with increased morbidity and mortality.^22^ Up to date, global concerns regarding acute respiratory infections (ARIs) still persist, and a noticeable increase in the incidence of infections caused by SARS-CoV-2 variants (JN.1, XEC, and KP.3 strains), IVs, human metapneumoviruses (HMPVs), and RSV has been observed during the post-COVID-19 era.^23^^,^^24^^,^^25^ Thus, understanding the infection profiles of diverse respiratory pathogens and their immune characteristics is critical for elucidating host-pathogen interactions, which can guide targeted disease prevention and control strategies.^26^

One essential aspect is the ability to assess the immunity of various respiratory pathogens within the context of an individual’s comprehensive immune landscape. A microarray platform allows proteome-wide profiling of antibody levels through high-throughput analysis from a single sample simultaneously, offering quantitative data about total immunoglobulins, as well as specific immunoglobulin responses.^27^^,^^28^^,^^29^ To date, the use of this microarray technology for parallel analysis of multiple respiratory pathogens is rare.

Herein, on the basis of a previously developed pan-respiratory pathogen (PRP) protein microarray (containing 56 respiratory pathogen strains), we comprehensively investigated immune responses to various pathogen infections and prior exposures in cohorts from different pandemic periods. Additionally, an independent post-pandemic cohort was used to confirm the reliability of the results. By evaluating humoral immune responses across the broader landscape of respiratory infections, our study offers valuable insights into the immunological correlates of protection and the durability of adaptive memory, thereby providing guidance for the optimization of strategies for addressing future respiratory pathogenic threats.

## Results

### Study cohorts and microarray performance validation

In this study, a total of 287 individuals, including 50 from the pre-pandemic period, 115 from the COVID-19 era, and 122 from the post-pandemic period, were included in the test cohort. Among them, 162 were males and 125 were females, with a median age of 47 years. In addition, an independent cohort comprising 175 individuals with SARS-CoV-2 infection from the post-pandemic period (2024–2025) was analyzed to validate the robustness of the results. The detailed information for both the test and validation datasets is presented in Table S2.

**Figure 1:**
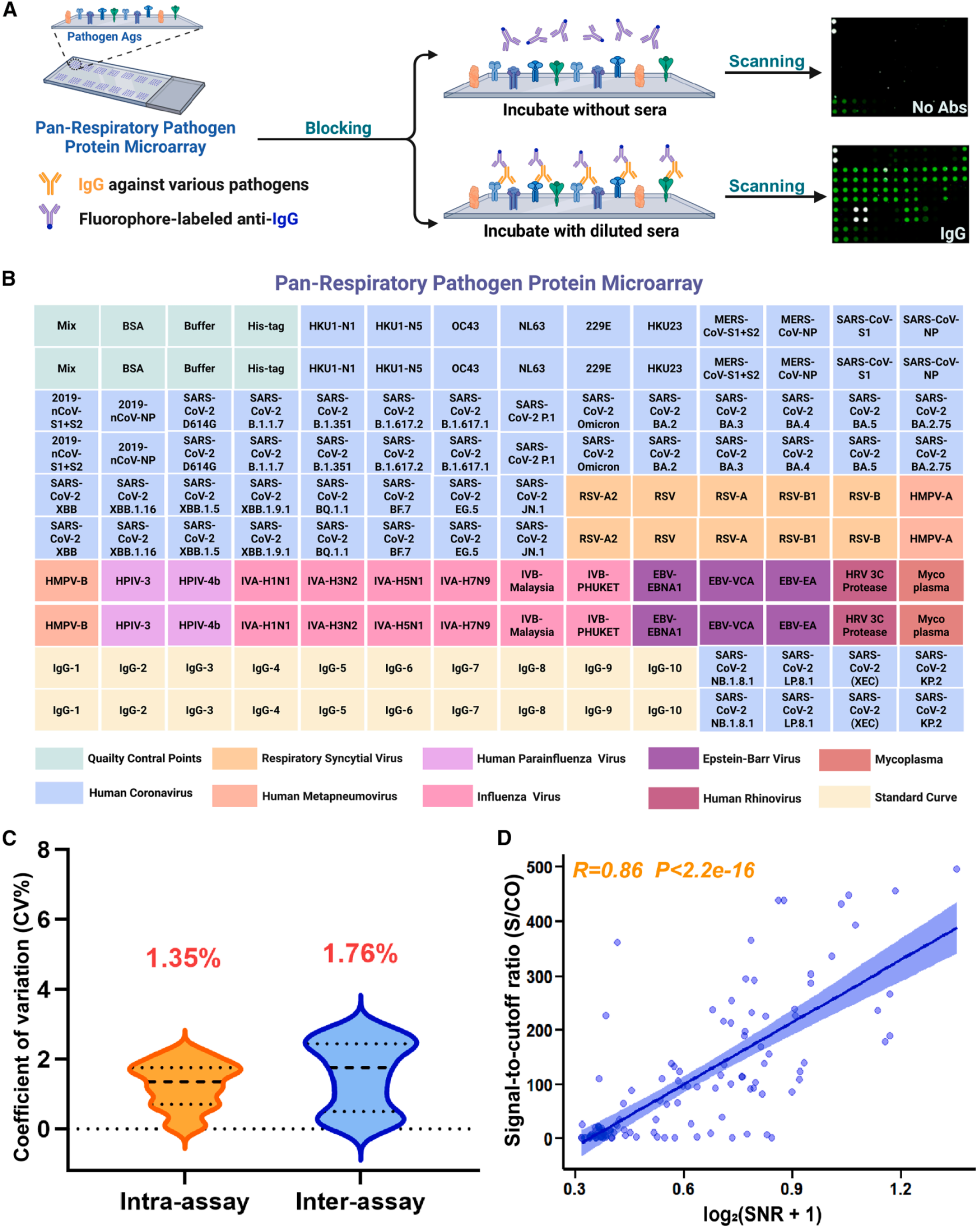
Development and analytical validation of the pan-respiratory pathogen protein microarray for IgG detection (A) Schematic of the IgG detection principle for the PRP protein microarray. (B) Layout of the main pathogen antigens (total 56 types) in the PRP protein microarray. (C) Intra- and inter-array of coefficients of variation (CVs) for the PRP protein microarray. In the violin plots, the bold dashed line represented the median, and the upper and lower nonbold lines represented the 75^th^ percentile (Q3) and the 25^th^ percentile (Q1), respectively. (D) Correlations of IgG responses with clinical detection results.

Herein, we utilized a previously developed high-throughput platform—a PRP protein microarray—to monitor immunoglobulin G (IgG) responses against PRP. A schematic of the two-step IgG signal detection is shown in Figure 1A; the principle is similar to that of the indirect enzyme-linked immunosorbent assay (ELISA), enabling specific differentiation of signal intensities from diverse antibodies using fluorescent-labeled secondary antibodies. The layout of 56 different pathogen protein antigens is listed in detail in Figure 1B, with each antigen printed in duplicate. Moreover, to evaluate the robustness and reproducibility of the platform, the PRP protein microarray exhibited remarkably low intra- and inter-array coefficients of variation (CVs) of 1.35% and 1.76%, respectively (Figure 1C). To further assess the reliability of the microarray strategy, Spearman correlation analysis was performed to compare the microarray-derived IgG values with signal-to-cutoff ratio (S/CO) obtained from the Clinical Laboratory of Beijing Ditan Hospital (Figure 1D). The correlation coefficient (*R*) of IgG was 0.86 (*p* < 0.001), indicating the strong feasibility of this microarray in practical clinical applications. Overall, these findings demonstrated that the PRP protein microarray exhibited highly reliable analytical performance.

### Comprehensive mapping of IgG reactivity profiles against highly pathogenic coronaviruses

**Figure 2:**
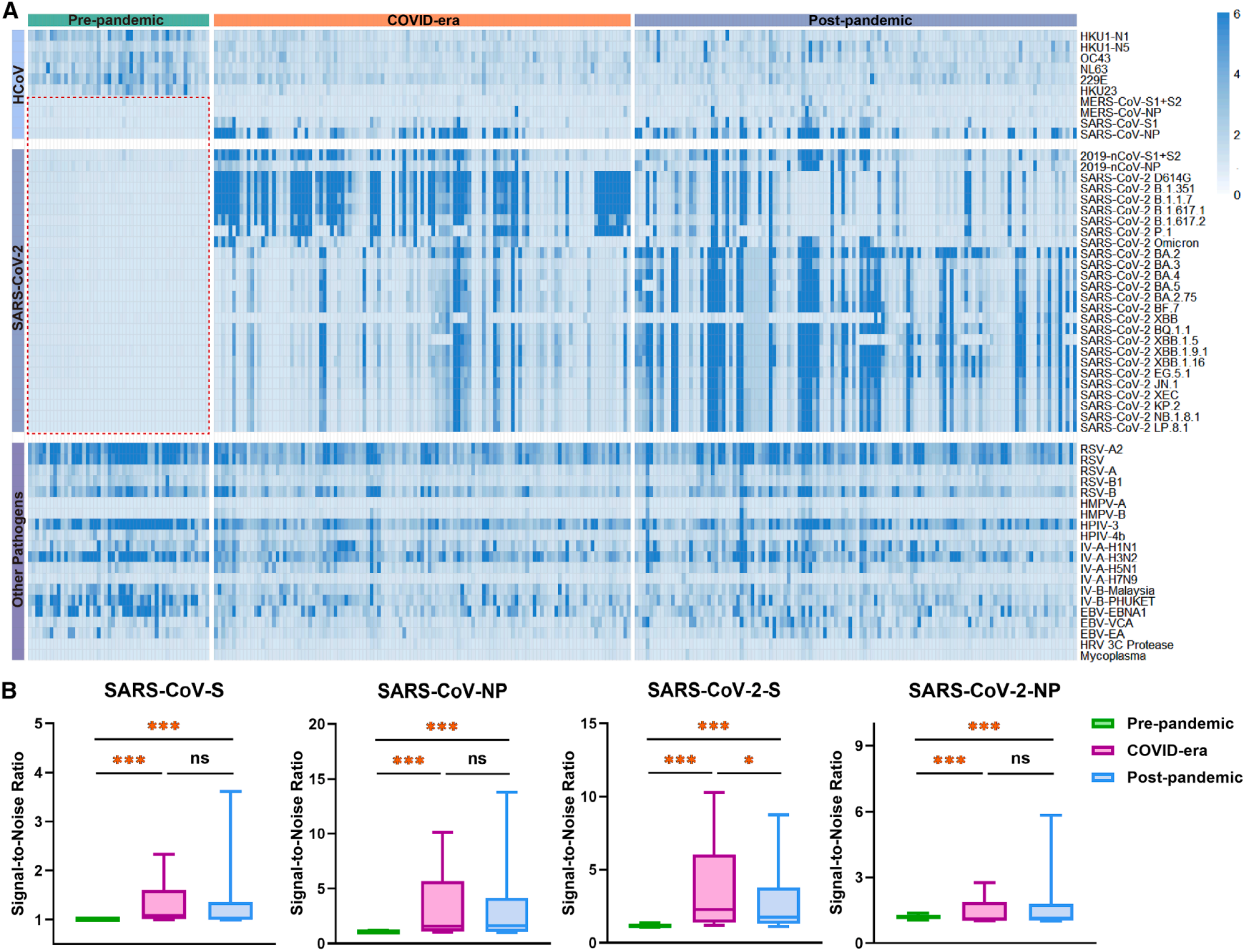
Respiratory pathogen-specific IgG profiles across the pre-pandemic, COVID-19 era, and post-pandemic cohorts (A) Profiling IgG microarray responses across distinct cohorts. This heatmap displayed the IgG reactivities against various respiratory pathogens across the three time groups, and the magnitudes of IgG response are reflected by color intensity. (B) IgG reactivity profiles against specific antigens of HPCoVs (SARS-CoV and SARS-CoV-2). Boxplots illustrated the distributions of the SNRs for the SARS-CoV spike (S), SARS-CoV nucleocapsid (NP), SARS-CoV-2-S, and SARS-CoV-2-NP proteins across the three periods. The boxes represented the interquartile range from the 25^th^ percentile (Q1) to the 75^th^ percentile (Q3), and the line within each box indicates the median. ∗*p* < 0.05, ∗∗*p* < 0.01, ∗∗∗*p* < 0.001, and ns, not significant.

To investigate the antibody profile associated with various pathogens, we evaluated differential IgG reactivity to specific antigens in three different cohorts. As depicted in Figure 2A, the total levels of IgG against HPCoVs (MERS-CoV, SARS-CoV, and SARS-CoV-2) were nearly undetectable in the pre-pandemic group, whereas those in the COVID-19 group and the post-pandemic cohort were markedly higher. These results revealed that there were no serological responses in the pre-pandemic population and further demonstrated the high specificity of the microarray platform without cross-reactivity. In particular, compared with the pre-pandemic baseline, the COVID-19 era and the post-pandemic cohort (*p* < 0.001) had significantly higher levels of IgG reactivity against the spike (S) and nucleocapsid (NP) proteins of both SARS-CoV-2 and SARS-CoV (Figure 2B). This finding highlights notable humoral immune responses and cross-reactivity between the two HPCoVs. However, the overall signal intensities of MERS-CoV antigens remained near the baseline level across all cohorts (Figure S1). The striking difference in cross-reactivity magnitude generally aligned with the phylogenetic relationship among these HPCoVs. Compared with the strong cross-reactivity to SARS-CoV, the negligible cross-reactivity to MERS-CoV reflects the lower homology between SARS-CoV-2 and MERS-CoV. Moreover, the IgG levels against SARS-CoV-2-NP remained relatively stable between the COVID-19 period and the post-pandemic period, whereas the reactivity against SARS-CoV-2-S significantly decreased in the post-pandemic cohort (*p* < 0.05). Given that NP-specific IgG is generally elicited by natural infection, its persistence highlights durable post-infection humoral memory, whereas the decrease in S-reactivity reflects the natural waning of both vaccine- and infection-induced antibodies over time.

**Figure 3:**
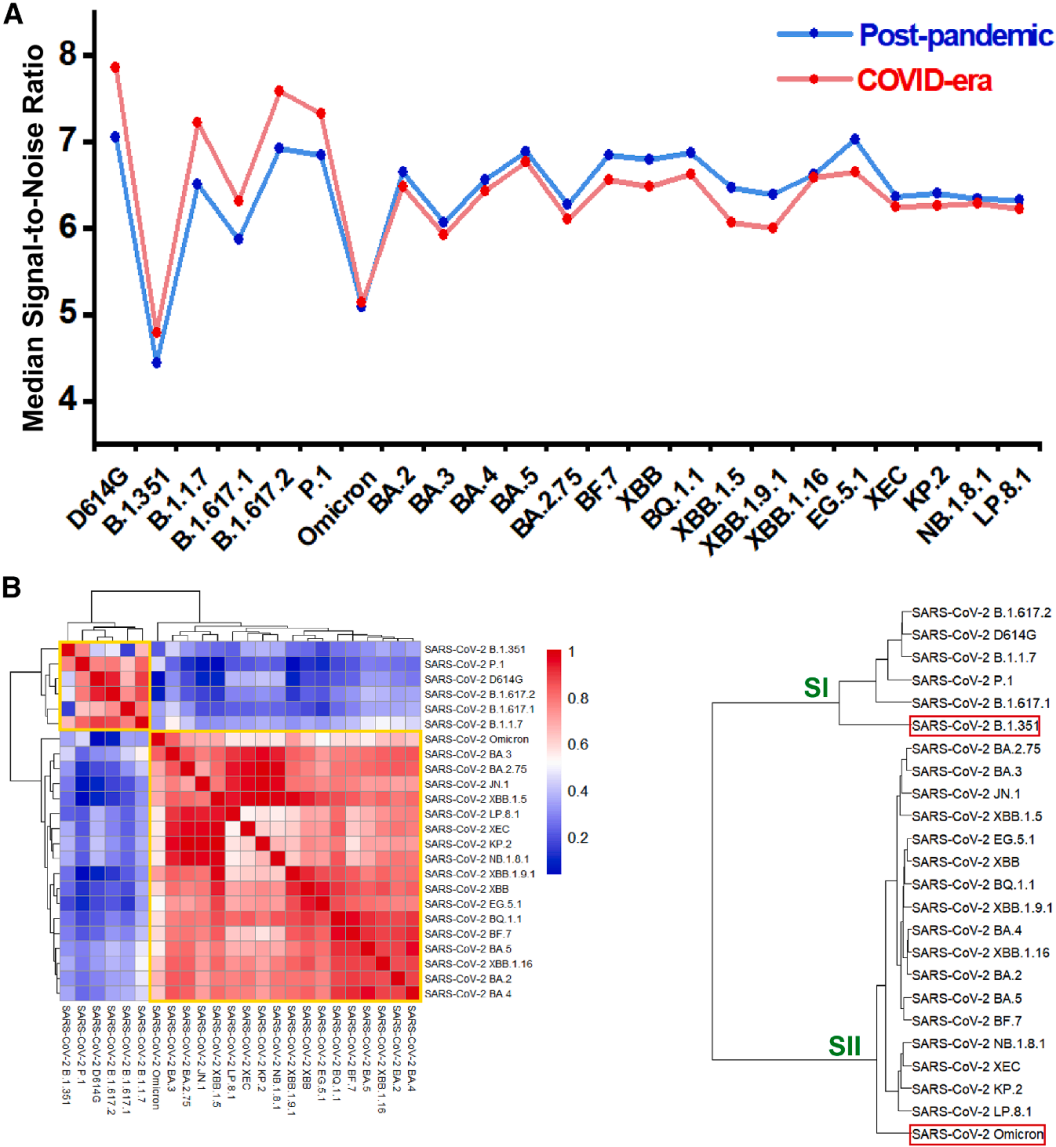
IgG responses to SARS-CoV-2 variants between the COVID-19 era and post-pandemic periods, and immunological classification of the variants (A) Line chart of IgG responses to SARS-CoV-2 variants during the COVID-19 era (red) and post-pandemic (blue) periods. (B) Immunological categorization of SARS-CoV-2 variants. Classification of SARS-CoV-2 serotypes was determined via IgG responses against the spike protein of the evaluated variant panel.

Furthermore, we also delineated the IgG reactivities during the COVID-19 era and the post-pandemic period and investigated broad-spectrum humoral immunity against diverse SARS-CoV-2 variants (Figure 3A). The analysis revealed a highly consistent trajectory of median signal-to-noise ratio (SNR) between the COVID-19 era and post-pandemic cohorts. Notably, dramatic declines in IgG binding were observed against the B.1.351 (Beta) and ancestral Omicron variants (B.1.1.529), highlighting their substantial immune escape ability. A distinct temporal inversion in antigen recognition was observed: during the COVID-19 era, the concentration of IgG against pre-Omicron strains was substantially greater than that against the Omicron lineage. Conversely, this pattern reversed during the post-pandemic period, when Omicron and its subvariants elicited stronger reactivities than the earlier strains did. This phenomenon highlights that Omicron variants remain the dominant strains in the present situation. In addition, we also classified SARS-CoV-2 variants according to the evolving antigenicity of their S protein. The classification of viral serotypes intrinsically depends on how divergent surface antigens drive differential humoral immune responses. All the variants were clustered into two different serotypes, as shown in Figure 3B, where serotype I (SI) represented pre-Omicron strains (D614G, B.1.351, B.1.1.7, B.1.617.1, B.1.617.2, and P.1), as well as serotype II (SII) comprising the Omicron lineage and its subvariants. Interestingly, the B.1.351 variant and ancestral Omicron variant formed distinct outlier branches. These findings corroborated the sharp declines in IgG reactivities observed earlier, further underscoring a trajectory of viral evolution driven by immune escape capabilities.

### Humoral immune landscapes against diverse pathogens across the study cohorts

**Figure 4:**
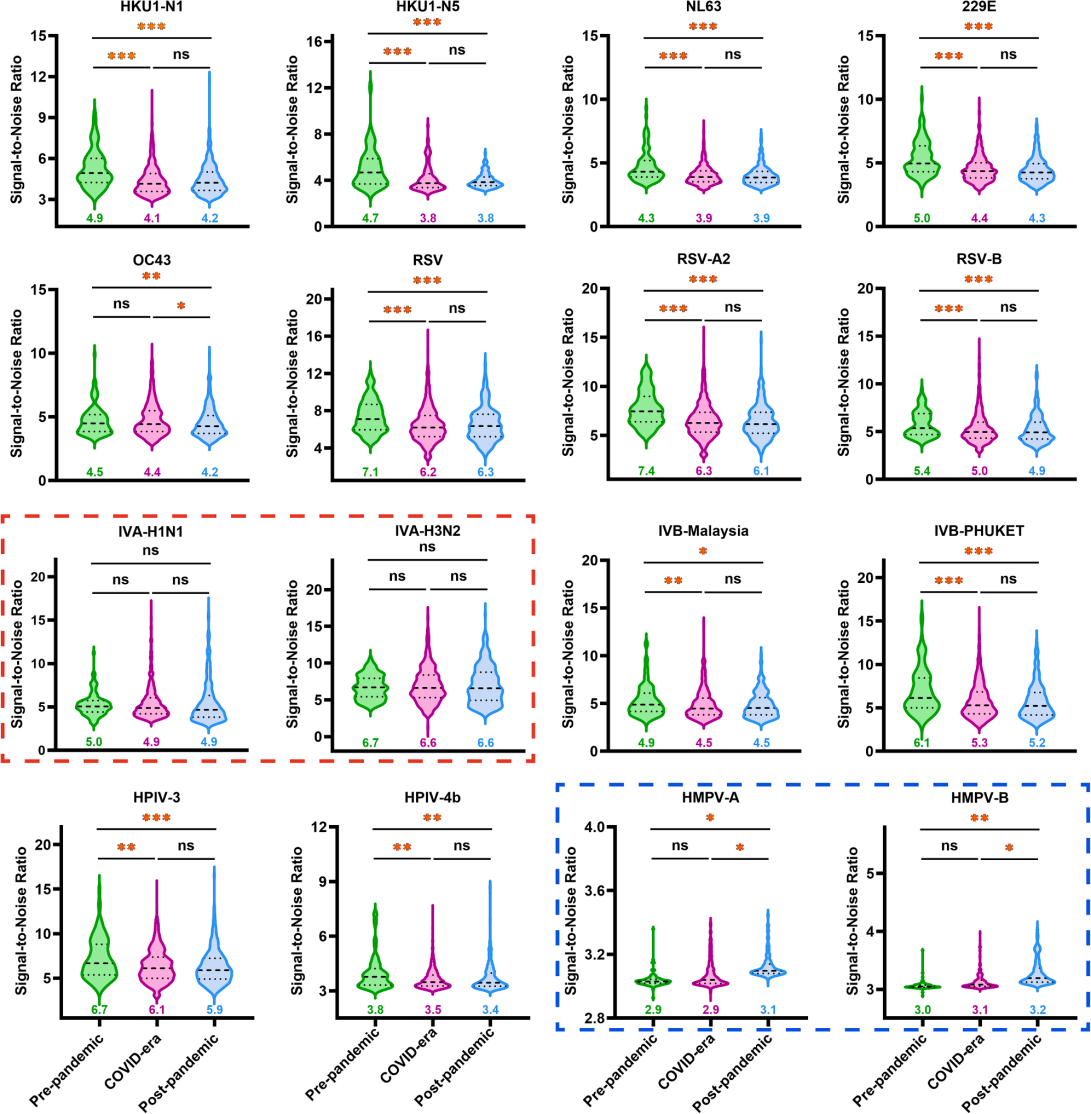
IgG reactivity profiles against pan-respiratory pathogens across pre-pandemic, COVID-19, and post-pandemic cohorts (prior to December 2023) Violin plots illustrating the SNRs of IgG targeting 16 respiratory viral antigens. The bold dashed line represented the median, and the upper and lower nonbold lines represented the 75^th^ percentile (Q3) and the 25^th^ percentile (Q1), respectively. ∗*p* < 0.05, ∗∗*p* < 0.01, ∗∗∗*p* < 0.001, and ns, not significant.

**Figure 5:**
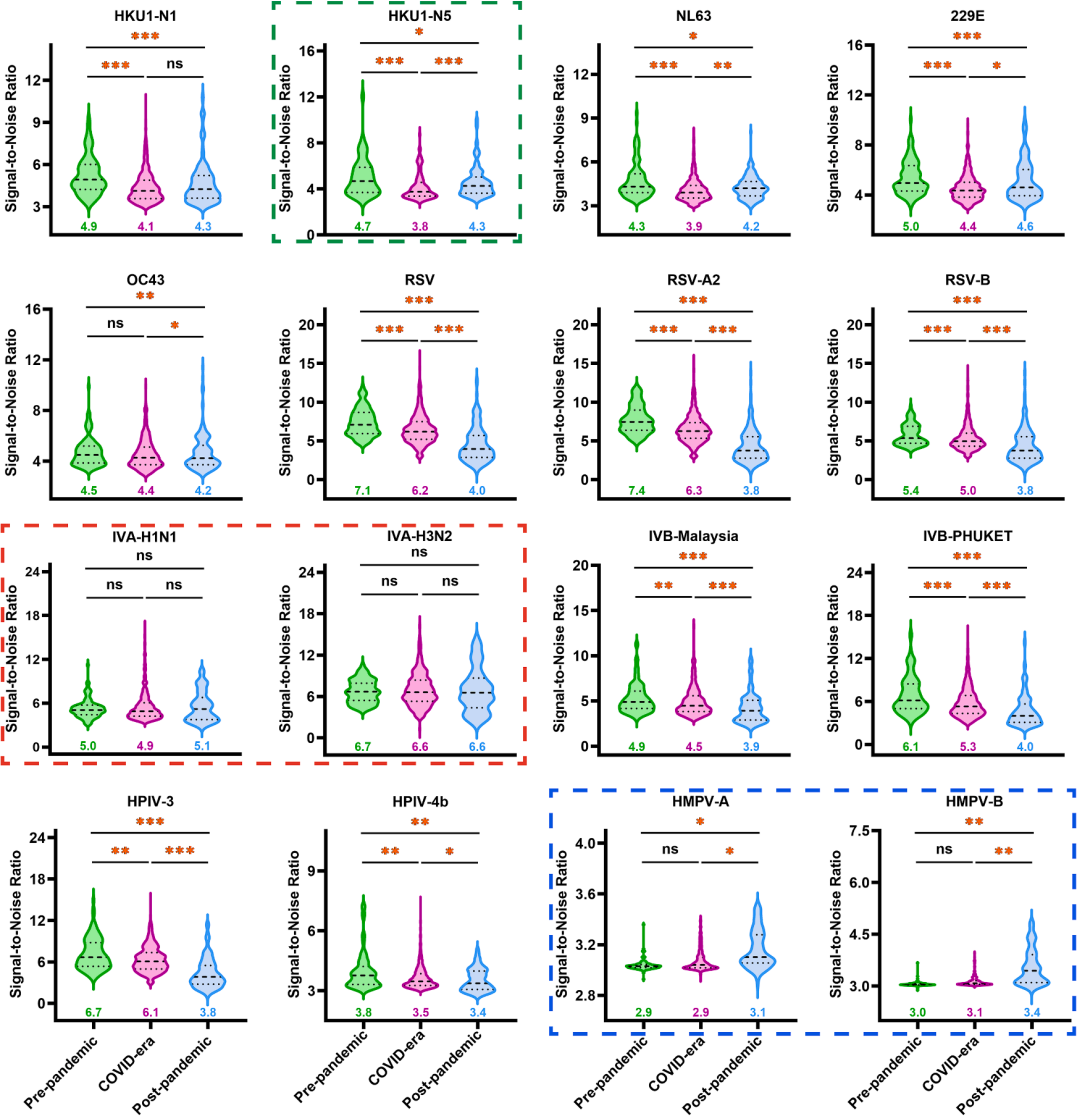
IgG reactivity profiles against pan-respiratory pathogens across the pre-pandemic, COVID-19, and post-pandemic cohorts (as an independent group for validation, 2024–2025) Violin plots illustrating the SNRs of IgG targeting 16 respiratory viral antigens. The bold dashed line represented the median, and the upper and lower nonbold lines represented the 75^th^ percentile (Q3) and the 25^th^ percentile (Q1), respectively. ∗*p* < 0.05, ∗∗*p* < 0.01, ∗∗∗*p* < 0.001, and ns, not significant.

The COVID-19 pandemic substantially altered the epidemiology of respiratory pathogens and reshaped humoral immune reactivity to pathogen infections. Therefore, we analyzed the response characteristics of IgG to elucidate the changes in population-level humoral immunity (Figure 4). Compared with the pre-pandemic baseline, the IgG intensities against the majority of common respiratory viruses, including HCoVs, RSVs, influenza virus B (IVB: Malaysia and Phuket), and human parainfluenza viruses (HPIVs), exhibited a ubiquitous and significant decline during the COVID-19 era and the post-pandemic periods. These observations aligned with the high prevalence of these pathogens among the general population, while rigorous NPIs were implemented during the COVID-19 era. In addition, the reactivities against influenza virus A (IVA: H1N1 and H3N2) remained stable during the pandemic period (red dashed box). Conversely, a unique and completely distinct pattern was observed for HMPV-A and HMPV-B (blue dashed box). IgG reactivities against HMPV significantly increased (*p* < 0.001) from the pre-pandemic baseline to the post-pandemic cohort. Furthermore, similar results were observed in an independent post-pandemic cohort (Figure 5). The specific IgG against H1N1 and H3N2 was still sustained at high titers, and HMPV reactivities continued to increase during the post-pandemic period (*p* < 0.001). In particular, the levels of HKU1-N5-specific IgG during post-pandemic era (green dashed box) exceeded their pre-pandemic level.

## Discussion

Given that the world has settled into the post-pandemic phase, clarifying the current state of immune activity against respiratory pathogens is important. The hybrid immune barrier has been established via high-coverage vaccination and natural infection to effectively attenuate clinical pathogenicity; however, the continuous selective pressure exerted by host immunity drives the adaptive evolution of SARS-CoV-2. In addition, NPIs and waning immune responses have the potential to change the epidemiological niches of traditional respiratory pathogens (especially HCoVs, RSVs, IVs, HPIVs, and HMPVs). Prolonged SARS-CoV-2 antigen exposure may subtly remodel the host’s preexisting immunological tone against other cocirculating pathogens through mechanisms of immune interference and viral competition. Thus, a post-pandemic era of the panoramic immunological landscape will provide an indispensable scientific foundation for formulating proactive intervention strategies.

As a core component of adaptive immunity, the humoral immune response shapes the profile of viral evolution and provides critical insights for epidemiological surveillance.^30^^,^^31^ Herein, our study represents the first effort to understand the relationship between immune memory in different cohorts, using well-defined serological characteristics to reflect pathogen-specific humoral responses. Moreover, we constructed a high-throughput microarray to simultaneously measure IgG responses targeting 56 respiratory pathogens, visualizing this paradigm shift in the population’s immunological landscape across three different cohorts (Figure 1). Among them, minimal HPCoV-specific reactivity was observed in the pre-pandemic cohort, indicating a lack of prior exposure to the virus prior to its global spread. This served as a vital baseline, ensuring that any identified reactivity in later groups was derived from SARS-CoV-2 infection or vaccination instead of cross-reactivity with preexisting coronaviruses (Figure 2A). The transition into the post-pandemic phase revealed that not only sustained immune responses against SARS-CoV-2 but also profound cross-reactive signatures against other HPCoVs.

As a member of the Betacoronavirus family, SARS-CoV-2 shares conserved epitopes with other HPCoVs, such as SARS-CoV (2002) and MERS-CoV (2012).^6^ While it is theoretically anticipated that cumulative exposure to SARS-CoV-2 might broaden the cross-reactive Ab repertoire, our findings revealed a highly specific and restricted cross-protection profile. Notably, repeated SARS-CoV-2 exposure induced a robust cross-reactive IgG against the closely related SARS-CoV (Figure 2B), but it failed to mount a substantial humoral response against heterologous MERS-CoV (Figure S1). This noticeable difference highlights the boundaries of the post-pandemic immunological repertoire, suggesting that the current hybrid immune system might offer vulnerable cross-protection against future spillovers of non-SARS-CoVs. In particular, IgG levels against SARS-CoV-2-S significantly decreased during the post-pandemic phase, which emphasized the decreasing nature of specific immunity.^32^ This decrease not only created a permissive environment for the continuous adaptive evolution of new variants but also validated the urgent need for ongoing seroepidemiological surveillance. By contrast, IgG responses to SARS-CoV-NPs demonstrated sustained durability, as they were closely associated with natural infection and might also be influenced by exposure to whole-virus inactivated vaccines. Although anti-NP antibodies may lack direct neutralizing ability, the persistent immunological memory they reflect plays a crucial role in mitigating disease severity.^33^^,^^34^

SARS-CoV-2 has undergone significant genetic evolution since it emerged in 2019. The evolution produced five major VOCs, namely, Alpha (B.1.1.7), Beta (B.1.351), Gamma (P.1), Delta (B.1.617.2), and Omicron (B.1.1.529), that have driven multiple surges of the global COVID-19 pandemic.^1^^,^^35^ Our study revealed that humoral immunity was predominantly focused on these VOCs in the COVID-19 era, while the immune response to Omicron and its subvariants markedly increased during the post-pandemic period (Figure 3A). This dynamic shift effectively reflected the evolution of population immunity following different SARS-CoV-2 exposures across pandemic phases. The COVID-19 humoral profile was predominantly shaped by early wild-type-based vaccinations and initial infections, whereas the post-pandemic landscape underscores an updated immune repertoire driven by widespread Omicron breakthrough infections. Consequently, this continuous antigen exposure significantly increased Omicron-specific cross-reactivity, whereas antibodies against the extinct pre-Omicron variants naturally decreased over time.^36^ Indeed, the transmission fitness of SARS-CoV-2 variants is governed by multifactorial determinants, including viral binding affinity to host receptors, immune evasion capability against antibodies, intrinsic viral replication efficiency, and host population immunity. These collective factors shape variant-specific epidemic trajectories and underscore the need for continuous surveillance of emerging variants.

Notably, our results also revealed a substantial decrease in IgG reactivities for B.1.351 and early Omicron, indicating that significant mutations in these strains allow them to effectively evade immune recognition in the population. These findings were further supported by the distinct branches observed in the serotypic classification (Figure 3B). Immune evasion in the Beta (B.1.351) variant was primarily driven by the E484K and K417N mutations in the receptor-binding domain (RBD).^37^ However, the Omicron (B.1.1.529) variant relies on extensive changes in the S protein, comprising more than 30 alterations that directly disrupt antibody recognition. Therefore, many mutations are supposed to have accumulated after prolonged viral adaptation within an immunocompromised host.^38^^,^^39^ Additionally, spike substitutions with immune escape effects, such as N501Y, could also be observed across these VOCs.^40^ Generally, SARS-CoV-2 employs multiple immune evasion strategies, including inhibiting IFN production, escaping the recognition of toll-like receptors (TLRs), modulating antigen presentation, manipulating cytokine signaling, and undergoing antigenic variation, all of which facilitate infection, promote inflammation, and reduce immune efficacy.^41^^,^^42^ Thus, emerging variants must be approached with caution as the disease transitions from a pandemic to an endemic phase.

In the post-pandemic era, the trajectories of immune reactivity against diverse respiratory pathogens exhibited marked divergence. The total levels of IgG against other respiratory pathogens (especially HCoVs, RSVs, IVB, and HPIVs) were lower during the pandemic period than during the pre-pandemic period (Figure 4). A plausible explanation for this observation was that strict NPIs (such as masking, area lockdowns, and school closures) were implemented during the COVID-19 period, which significantly reduced interpersonal contact and simultaneously interrupted the transmission of these endemic respiratory pathogens.^43^^,^^44^^,^^45^^,^^46^ Thus, while the disruption in viral exposure resulted in a decrease in recurrent infection outbreaks, it concurrently led to decreasing IgG levels with prior infections. Moreover, this immunity gap necessitates increased vigilance for vulnerable populations, particularly the elderly people and children. In contrast, the IgG levels of HMPVs clearly increased during the post-pandemic period. NPIs altered HMPV’s normal transmission dynamics, which might have created a potential immunity debt. As a result, following the relaxation of restrictions, increased close contact triggered a marked rebound in infections. Furthermore, HMPV circulation was not entirely suppressed during the pandemic, leading to ongoing asymptomatic infections that contributed to IgG accumulation. Another possibility is that immune responses to SARS-CoV-2 infection or vaccination unexpectedly produced IgG with partial cross-neutralizing activity against HMPVs; however, the exact underlying cause or mechanism remains to be further explored. Moreover, these findings were independently validated in a distinct group, and the trend of HKU1-N5 was comparable to that of HMPV in the independent validation set (Figure 5).

Notably, compared with the pre-pandemic period, the reactivities against IVA (H1N1 and H3N2) remained stable during the post-pandemic period, suggesting that these strains sustained transmission and immune recognition during the COVID-19 era.^47^ Fundamentally, SARS-CoV-2 and IVA infections likely do not exhibit strong competition, as their distinct fusion mechanisms and regulatory networks reduce direct competition for host resources.^48^ A study also revealed that the biological and replicative dynamics of IVA remained unperturbed by SARS-CoV-2 infection, irrespective of the temporal sequence or interval between exposures, indicating limited reciprocal interference.^49^ As expected, coinfections with COVID-19 and influenza have been widely identified and reported, especially after easing COVID-19 restrictions.^50^^,^^51^^,^^52^ Another reason for these differences in the immune response to IVA might depend on reactions to past exposure and vaccinations. Individuals who have been previously exposed to IVA sometimes exhibit enhanced viral replication.^53^ Consequently, the COVID-19 pandemic has reshaped the immune status against respiratory pathogens and even the entire level of human immunity in the studied cohorts; however, further studies are needed.

In summary, we profiled the humoral immune responses of cohort populations across distinct time periods on the basis of the developed PRP protein microarray. Our analysis revealed that the COVID-19 pandemic noticeably shaped the SARS-CoV-2-related immune landscape, forming a specific trajectory of evolution and modulating the adaptation of immune responses. In addition, the COVID-19 era also affected the patterns of immune memory across common respiratory pathogens, potentially influencing susceptibility and protection against respiratory infections in future. Overall, this comprehensive immunological mapping provides critical insights into the evolving landscape of respiratory infections and epidemiological surveillance in the post-pandemic era.

### Limitations of the study

Several limitations of this work need to be acknowledged. First, the restricted samples and small-scale cohort may have caused statistical bias, and residual variation related to demographic factors such as age and sex cannot be completely excluded. Larger populations and evaluations across individuals with other infection conditions are needed. Second, it is necessary to conduct comprehensive analyses covering the complete viral proteome and correlating long-term immune responses in respiratory infections; thus, the sera of individuals with follow-up data should be collected for further study. In addition, vaccination status, booster history, and the timing of SARS-CoV-2 infection were not incorporated into the present analysis, but they were also important factors that influenced antibody responses at the individual level. Third, although the microassay currently features a broad pathogen panel, it lacks comprehensive coverage of circulating strains.

## Resource availability

### Lead contact

Requests for further information and resources should be directed to the lead contact, Yajie Wang (wangyajie@ccmu.edu.cn).

### Materials availability

This study did not generate new unique reagents.

### Data and code availability

• Data reported in this paper will be shared by the lead contact upon request.
• This paper does not report original code.
• Any additional information required to reanalyze the data reported in this paper is available from the lead contact upon request.

## Acknowledgments

This work was supported by the 10.13039/501100012166National Key Research and Development Program of China (2022YFE0210400), Beijing Municipal Natural Science Foundation (M21003), and Beijing Hospitals Authority’s Ascent Plan (DFL20241801).

## Author contributions

X.X. and H.W., investigation, writing – original draft, and methodology; L.T., methodology and writing – review and editing; P.N. and F.Y., writing – review, editing, investigation, and formal analysis; Y.W., review, supervision, and funding acquisition.

## Declaration of interests

The authors declare that there are no competing conflicts of interest.

## STAR★Methods

### Key resources table

**Table:** 

REAGENT or RESOURCE	SOURCE	IDENTIFIER
**Antibodies**		

Cy3-Donkey-*anti*-human-IgG (H + L)	Jackson ImmunoResearch	Cat# 709-165-149; RRID: AB_2340535

**Chemicals, peptides, and recombinant proteins**		

PBST (0.05% [v/v] Tween 20)	Solarbio, Beijing, China	P1031
Bovine Serum Albumin, BSA	Sigma-AldrichTrading Co., Ltd., Shanghai, China	Cat# A7906

**Critical commercial assays**		

GenePix 4300A microarray scanner	Molecular Devices, Sunnyvale, CA, USA	N/A

**Deposited data**		

Raw and processed pan-respiratory pathogen protein microarray data	This paper	N/A

**Software and algorithms**		

R software (version 4.4.1)	R Foundation for Statistical Computing, Vienna, Austria	https://www.r-project.org/
LuxScan 10K software	CapitalBio Corp., Beijing, China	N/A

### Experimental model and study participant details

#### Study participants and ethical approval

A total of 287 individuals were involved in this study, which was classfied into three cohorts: pre-pandemic (*n* = 50), COVID-era (*n* = 115), and post-pandemic cohort (*n* = 122, prior to December 2023). The pre-pandemic cohort comprised serum samples collected before December 2019 without SARS-CoV-2 infection. COVID-era was the “Dynamic Zero-COVID Policy” (DZCP) period in China from December 2019 to December 2022, and the post-pandemic period was refered to after December 2022, following the substantial relaxation of COVID-19 restrictions by the official governmen. These time frames were selected to capture the transition from the pre-pandemic baseline to the period of intensive public health interventions and then to the post-restriction phase. Both COVID-era and post-pandemic cohorts included serum samples collected from individuals naturally infected with SARS-CoV-2 or health check-ups. Furthermore,we further validated these findings in an independent cohort comprising 175 SARS-CoV-2 cases during the post-pandemic period (2024–2025). It was used to assess the consistency of major humoral immune-reactivity patterns between the early post-pandemic cohort and a later post-pandemic population.

This study was also approved by the Ethics Committee of Beijing Ditan Hospital (Nos. DTEC-KY2022–052-02 and DTEC-KY2021–010-01). Sera samples were covered by the approved study protocol, and written informed consent was obtained from all participants before sera collection. All procedures strictly adhered to the principles of the Declaration of Helsinki.

### Method details

#### Specimen collection and storage

All serum samples were obtained from the Department of Clinical Laboratory, Beijing Ditan Hospital. As for sample collection processes, whole blood was initially collected into vacutainer tubes, and centrifuged at 4,000×g for 10 min to isolate the upper serum. Finally, the separated serum was transferred to storage tubes, and stored at −80 °C until experiments.

#### Design of the PRP protein microarrays

The Pan-respiratory pathogens (PRP) protein microarray was constructed based on previously reported method with slight modification,^29^ mainly involving slide pretreatment, antigen preparation, and antigen protein immobilization. Meanwhile, the reliability of the modified procedure was assessed by intra- and inter-array reproducibility analyses and comparison with routine clinical detection results. We focused on the antigens about human coronaviruses (HCoVs: HKU1-N1, HKU1-N5, OC43, NL63, 229E and HKU23), SARS-CoV-2 with its major variants, other HPCoVs (SARS-CoV and MERS-CoV), and 20 types of non-coronavirus respiratory pathogens (RSVs, HMPVs, IVs, HPIVs, HRVs and mycoplasma). These selected antigens were representative of different respiratory pathogens and were chosen based on their specificity to the corresponding pathogen. In particular, the criteria for antigen selection primarily included specificity (without cross-reactivity), binding affinity to the target antibody, and characteristic conducive to overcoming multiplex matrix effects. All of the purified pathogen antigens were purchased from Jackson ImmunoResearch, and the detailed antigen information was summarized in Table S1.

#### Detection of IgG via the PRP protein microarrays

Prior to processing, the PRP protein microarrays were allowed to equilibrate to room temperature for 30 min. To prevent non-specific binding, the microarrays were blocked with 5% v/v milk in PBST (0.05% v/v Tween 20 in PBS) for 1 h. After blocking, the microarrays were incubated with the 5000-fold diluted sera in 5% v/v milk for 1 h on shaking incubator. Followed by washing again, the arrays were incubated for 0.5 h with secondary antibody, 2.0 μg/mL Cy3-Donkey-*anti*-human-IgG (H + L) (Jackson ImmunoResearch). Finally, the microassays were scanned using a GenePix 4300A microarray scanner (Molecular Devices, Sunnyvale, CA, USA) at 532 nm. In addition, the median fluorescent signal intensity (MFI) was extracted through the LuxScan 10K software (CapitalBio Corp., Beijing, China), and the median spot signal intensity was corrected by subtracting the median background signal.

### Quantification and statistical analysis

#### Microarray signal quantification and normalization

For each spot, the raw fluorescence intensity was obtained by subtracting the median background intensity from the median signal intensity, and the resulting values were averaged across duplicates. Then normalization was performed using the signal-to-noise ratio (SNR), which was obtained by dividing the mean signal by the 25^th^ percentile intensity of the negative control spots as background. The detailed calculation formula was as follows:

$${SNR}_{\;Spot}=\frac{\;I_{spot}}{\;I_{background}}$$

where *I*_*spot*_ represented the average fluorescence intensity of the specific spot printed in duplicate, and *I*_*background*_ was defined as the 25% quantile of the fluorescence intensity obtained from the duplicate negative control spots.

### Statistical analysis

Intra- and inter-array coefficients of variation (*CVs*) were calculated to assess the repeatability and reproducibility of the PRP protein microarray, respectively. Correlation coefficient (*R*) between microarray-based IgG measurements and clinical assay results were assessed using two-sided Spearman analysis. Then the Shapiro–Wilk test was used to assess data normality, and comparisons across multiple groups were performed using the Kruskal–Wallis test, followed by Dunn’s test for post hoc pairwise comparisons. Statistical significance was determined via Bonferroni-adjusted *p*-values (<0.05).

Furthermore, R software (version 4.4.1) was utilized for all the data analyses. Statistical significance levels were indicated as ∗*p* < 0.05, ∗∗*p* < 0.01, ∗∗∗*p* < 0.001, ns, no significance.

## Supplemental information

Document S1. Figure S1 and Tables S1 andS2
