# Supplementary material for: Comprehensive profiling of humoral immune responses to pan-respiratory pathogens: Insights from diverse cohorts
Source: iScience. 2026 Jul 31;29(8):117050. doi: 10.1016/j.isci.2026.117050 (PMC13452239; doi:10.1016/j.isci.2026.117050)
Supplement: Supplementary file 1 [file mmc1.pdf]

iScience, Volume *29*

Supplemental information

Comprehensive profiling of humoral immune responses to pan-respiratory pathogens: Insights from diverse cohorts

Xuanming Xu, Hui Wang, Lian Tang, Peiru Nie, Fengzhou Yang, and Yajie Wang

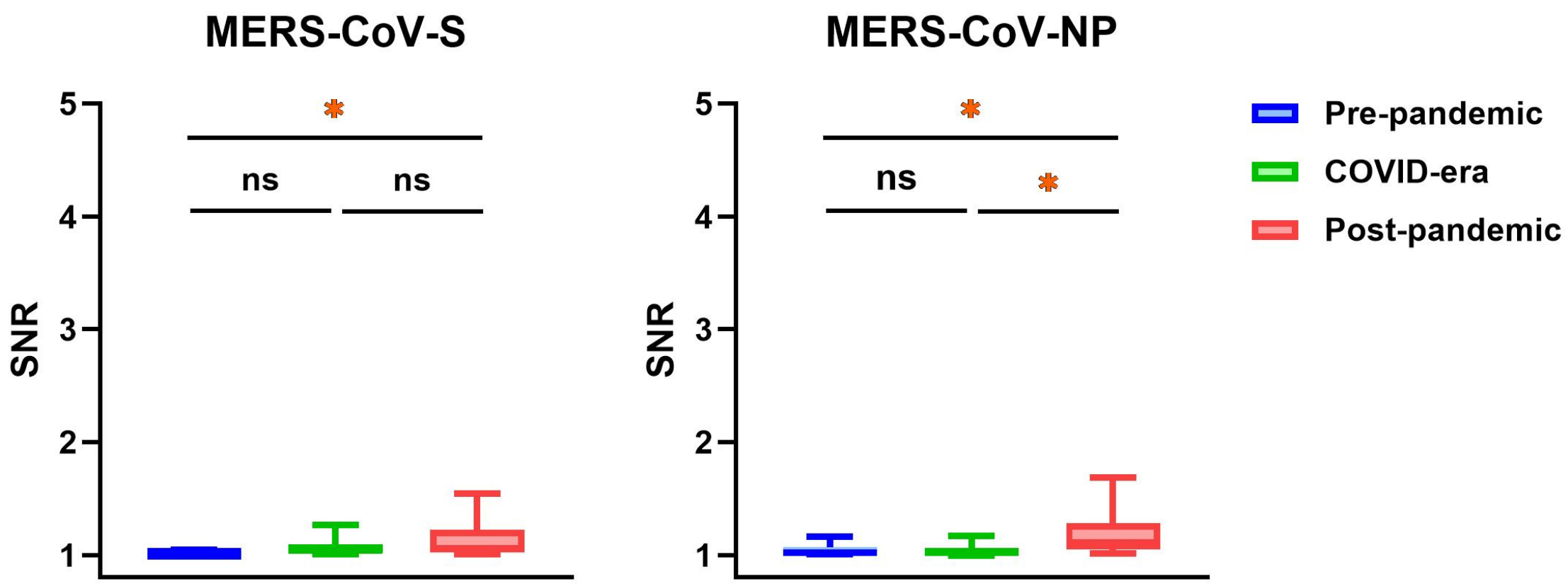

Figure S1. IgG reactivity profiles against specific antigens of MERS-CoV. Box plots illustrated the distribution of SNR values for MERS-CoV spike (S), MERS-CoV nucleocapsid protein (NP) across the three periods. The boxes represented the interquartile range from the 25th percentile (Q1) to the 75th percentile (Q3), and the line within each box indicates the median. $^{*}P<0.05$; $^{**}P<0.01$; $^{***}P<0.001$; ns, not significant.

Table S1 Antigen names and their detailed information.

Column	Row	Full name of protein on the array	Short name of protein on the array	
1	1	Human IgG+IgM（200 ug/mL）	Mix	
2	1	BSA（200 ug/mL）	BSA	
3	1	PBS	Buffer	
4	1	6His（200 ug/ml）	His-Tag	
5	1	HKU1 (isolate N1) (HCoV-HKU1) Spike/S1 Protein (S1 Subunit, His Tag)	HKU1-N1	P1
6	1	HKU1 (isolate N5) (HCoV-HKU1) Spike Protein (S1+S2 ECD, His Tag)	HKU1-N5	P2
7	1	(HCoV-OC43) Spike Protein (S1+S2 ECD, His Tag)	OC43	P3
8	1	(HCoV-NL63) Spike Protein (S1+S2 ECD, His Tag)	NL63	P4
9	1	(HCoV-229E) Spike Protein (S1+S2 ECD, His Tag)	229E	P5
10	1	(DcCoV)(strain HKU23-368F) Nucleoprotein / DcCoV-NP Protein (His Tag)	HKU23	P6
11	1	MERS-CoV Spike Protein (S1+S2 ECD, aa 1-1297, His Tag)	MERS-CoV-S1+S2	P7
12	1	MERS-CoV Nucleocapsid Protein (His Tag)	MERS-CoV-NP	P8
13	1	SARS-CoV Spike/S1 Protein (S1 Subunit, His Tag)	SARS-CoV-S1	P9
14	1	SARS-CoV Nucleocapsid Protein (His Tag)	SARS-CoV-NP	P10
1	3	SARS-CoV-2 (2019-nCoV) Spike S1+S2 ECD-His Recombinant Protein	2019-nCoV-S1+S2	P11
2	3	SARS-CoV-2 (2019-nCoV) Nucleocapsid-His recombinant Protein	2019-nCoV-NP	P12
3	3	SARS-CoV-2 Spike S1+S2 (D614G) trimer Protein (ECD, His tag) (HPLC-verified)	SARS-CoV-2 D614G	P13
4	3	SARS-CoV-2 B.1.1.7 Spike S1+S2 trimer Protein (ECD, His tag) (HPLC-verified)	SARS-CoV-2 B.1.1.7	P14
5	3	SARS-CoV-2 B.1.351 Spike S1+S2 trimer Protein (ECD, His tag) (HPLC-verified)	SARS-CoV-2 B.1.351	P15
6	3	SARS-CoV-2 B.1.617.2 Spike S1+S2 trimer Protein (ECD, His tag) (HPLC-verified)	SARS-CoV-2 B.1.617.2	P16
7	3	SARS-CoV-2 B.1.617.1 Spike S1+S2 trimer Protein (ECD, His tag) (HPLC-verified)	SARS-CoV-2 B.1.617.1	P17
8	3	SARS-CoV-2 (P.1) Spike S1+S2 trimer Protein (ECD, His tag) (HPLC-verified)	SARS-CoV-2 P.1	P18
9	3	SARS-CoV-2 B.1.1.529 (Omicron) S1+S2 trimer Protein (ECD, His Tag) (HPLC-verified)	SARS-CoV-2 Omicron	P19
10	3	SARS-CoV-2 (BA.2) Spike S1+S2 trimer Protein (ECD, His Tag) (HPLC-verified)	SARS-CoV-2 BA.2	P20
11	3	SARS-CoV-2 Spike Trimer Protein, His Tag (BA.3/Omicron) (MALS verified)	SARS-CoV-2 BA.3	P21

12	3	SARS-CoV-2 Spike Trimer Protein, His Tag (BA.4/Omicron) (MALS verified)	SARS-CoV-2 BA.4	P22
13	3	SARS-CoV-2 Spike Trimer Protein, His Tag (BA.5/Omicron) (MALS verified	SARS-CoV-2 BA.5	P23
14	3	SARS-CoV-2 (BA.2.75) Spike S1+S2 trimer Protein (ECD, His Tag) (HPLC-verified)	SARS-CoV-2 BA.2.75	P24
1	5	SARS-CoV-2 XBB (Omicron) Spike S1+S2 trimer Protein (ECD, His Tag) (HPLC-verified)	SARS-CoV-2 XBB	P25
2	5	SARS-CoV-2 XBB.1.16 (Omicron) Spike S1+S2 trimer Protein (ECD, His Tag) (HPLC-verified)	SARS-CoV-2 XBB1.16	P26
3	5	SARS-CoV-2 XBB.1.5 (Omicron) Spike S1+S2 trimer Protein (ECD, His Tag) (HPLC-verified)	SARS-CoV-2 XBB.1.5	P27
4	5	SARS-CoV-2 XBB.1.9.1 (Omicron) Spike S1+S2 trimer Protein (ECD,His Tag)(HPLC-verified)	SARS-CoV-2 XBB.1.9.1	P28
5	5	SARS-CoV-2 BQ.1.1 (Omicron) Spike S1+S2 trimer Protein (ECD, His Tag) (HPLC-verified)	SARS-CoV-2 BQ.1.1	P29
6	5	SARS-CoV-2 BF.7 (Omicron) Spike S1+S2 trimer Protein (ECD, His Tag) (HPLC-verified)	SARS-CoV-2 BF.7	P30
7	5	SARS-CoV-2 EG.5.1 S-trimer	SARS-CoV-2 EG.5.1	P31
8	5	SARS-CoV-2 JN.1 S-trimer	SARS-CoV-2 JN.1	P32
9	5	(RSV) (A2) Fusion glycoprotein / RSV-F Protein (ECD, His Tag)	RSV-A2	P33
10	5	(RSV) Fusion protein / RSV-F (Strain RSS-2) Protein (His Tag)	RSV	P34
11	5	(RSV) (A, rsb1734) glycoprotein G / RSV-G Protein (95% Homology) (His Tag)	RSV-A	P35
12	5	RSV (B1) glycoprotein G / RSV-G Protein (His Tag)	RSV-B1	P36
13	5	(RSV) (B, strain 18537) Fusion glycoprotein F0 / RSV-F Protein (His Tag)	RSV-B	P37
14	5	Human metapneumovirus (strain HMPV/Homo sapiens/PER/CFI0320/2010/A) glycoprotein G Protein (His Tag)	HMPV-A	P38
1	7	Human metapneumovirus (strain HMPV/Homo sapiens/PER/CFI0466/2010/B) glycoprotein G Protein (His Tag)	HMPV-B	P39
2	7	(HPIV-3) (strain Wash/47885/57) Hemagglutinin-neuraminidase Protein (His Tag)	HPIV-3	P40
3	7	(HPIV-4b) Hemagglutinin-neuraminidase Protein (aa 48-579, His Tag)	HPIV-4b	P41
4	7	Influenza A H1N1 (A/Beijing/22808/2009) Hemagglutinin / HA Protein (His Tag)	IV-A-H1N1	P42
5	7	Influenza A H3N2 (A/Texas/50/2012) Hemagglutinin / HA Protein (His Tag)	IV-A-H3N2	P43

6	7	Influenza A H5N1 (A/VietNam/1203/2004) Hemagglutinin / HA Protein (His & Fc Tag)	IV-A-H5N1	P44
7	7	Influenza A H7N9 (A/Anhui/1/2013) Hemagglutinin / HA Protein (His Tag)	IV-A-H7N9	P45
8	7	Influenza B (B/Malaysia/2506/2004) Hemagglutinin / HA Protein (His Tag)	IV-B-Malaysia	P46
9	7	Influenza B (B/PHUKET/3073/2013) Hemagglutinin / HA Protein (His Tag)	IV-B-PHUKET	P47
10	7	EBV-EBNA1	EBV-EBNA1	P48
11	7	EBV-VCA	EBV-VCA	P49
12	7	EBV-EA	EBV-EA	P50
13	7	Recombinant HRV 3C Protease	HRV 3C Protease	P51
14	7	Mycoplasma protein	Mycoplasma	P52
11	9	SARS-CoV-2 NB.1.8.1 S-trimer	SARS-CoV-2 NB.1.8.1	P53
12	9	SARS-CoV-2 LP.8.1 S-trimer	SARS-CoV-2 LP.8.1	P54
13	9	SARS-CoV-2 XEC S-trimer	SARS-CoV-2 XEC	P55
14	9	SARS-CoV-2 KP.2 S-trimer	SARS-CoV-2 KP.2	P56

Table S2 Baseline characteristics of the study cohorts.

**Cohort**	**Various period**	**Median Age (year)**		**Sex**		**Total Number**
				**Male**	**Female**	
**Test**	Pre-pandemic	45 (15-87)	SARS-CoV-2 (-)	23	27	50
	COVID-era	40 (15-87)	SARS-CoV-2 (+)	65	50	115
	Post-pandemic	60 (20-89)	SARS-CoV-2 (+)	74	48	122
		47 (15-89)		162	125	287
**Validation**	Post-pandemic	52 (14-95)	SARS-Cov-2 (+)	105	70	175
